# The structural influence of family and parenting on young people's sexual and reproductive health in rural northern Tanzania

**DOI:** 10.1080/13691058.2014.992044

**Published:** 2015-01-19

**Authors:** Joyce Wamoyi, Daniel Wight, Pieter Remes

**Affiliations:** ^a^ National Institute for Medical Research, Mwanza, Tanzania; ^b^ Medical Research Council, Social and Public Health Sciences Unit, Glasgow, UK

**Keywords:** parenting, structural factors, socialisation, young people, sexual and reproductive health, Africa

## Abstract

This paper explores the structural role of the family and parenting in young people's sexual and reproductive health. The study involved eight weeks of participant observation, 26 in-depth interviews, and 11 group discussions with young people aged 14–24 years, and 20 in-depth interviews and 6 group discussions with parents/carers of children in this age group. At an individual level, parenting and family structure were found to affect young people's sexual behaviour by influencing children's self-confidence and interactional competence, limiting discussion of sexual health and shaping economic provision for children, which in turn affected parental authority and daughters' engagement in risky sexual behaviour. Sexual norms are reproduced both through parents' explicit prohibitions and their own behaviours. Girls are socialised to accept men's superiority, which shapes their negotiation of sexual relationships. Interventions to improve young people's sexual and reproductive health should recognise the structural effects of parenting, both in terms of direct influences on children and the dynamics by which structural barriers such as gendered power relations and cultural norms around sexuality are transmitted across generations.

## Abstract (es)

En este artículo se analiza qué función estructural desempeñan la familia y los padres en cuanto a la salud sexual y reproductiva de los jóvenes. En este estudio de 8 semanas de duración se observó a los participantes mediante 26 entrevistas exhaustivas y 11 charlas en grupo con jóvenes con edades comprendidas entre 14 y 24 años, y 20 entrevistas exhaustivas y 6 charlas en grupo con padres y cuidadores de menores de este grupo de edad. A nivel individual se observó que la estructura parental y familiar afectaba a la conducta sexual de los jóvenes porque influía en su autoestima y competencia interaccional, limitaba el debate sobre la salud sexual, y daba forma a la prestación económica para menores, lo que a su vez afectaba a la autoridad parental y la participación de las hijas en conductas sexuales arriesgadas. Las normas sexuales se reproducen a través de las prohibiciones explícitas de los padres y sus propias conductas. Se enseña socialmente a las chicas a aceptar la superioridad masculina, lo que determinará su modo de negociar las relaciones sexuales. En los programas para mejorar la salud sexual y reproductiva de los jóvenes se deberían reconocer los efectos estructurales de los padres, ya sea por la influencia directa de los padres en los hijos o por las dinámicas por las que los obstáculos estructurales, tales como las relaciones de poder masculino y las normas culturales en torno a la sexualidad, se transmiten de una generación a otra.

## Abstract (fr)

Cette étude a examiné l'impact structurel de la famille et des compétences parentales sur la santé sexuelle et reproductive des jeunes. Elle a consisté en 8 semaines d'observation participative, 26 entretiens en profondeur et 11 groupes de discussion thématique avec des jeunes âgés de 14 à 24 ans; et en 20 entretiens en profondeur et 6 groupes de discussion thématique avec des parents/responsables d'enfants dans cette tranche d'âge. L'étude à révélé qu'au niveau individuel, les compétences des parents et la structure familiale avaient un impact sur les comportements sexuels des jeunes en influençant la confiance en soi et les compétences interactionnelles chez les enfants; en restreignant les discussions sur la santé sexuelle; et en déterminant le soutien économique pour les enfants qui, en retour, impactait l'autorité parentale et l'engagement des filles dans des comportements sexuels à risque. Les normes sexuelles sont reproduites à la fois à travers les interdictions explicites des parents et les comportements de ces mêmes parents. Les filles sont socialisées pour accepter la supériorité masculine, ce qui détermine la manière avec laquelle elles négocient les rapports sexuels. Les interventions visant à améliorer la santé sexuelle et reproductive chez les jeunes doivent reconnaître l'impact structurel des compétences parentales, aussi bien par rapport à leur influence directe sur les enfants que par rapport à la dynamique selon laquelle les obstacles structurels, comme les relations de pouvoir fondées sur le genre et les normes culturelles de la sexualité, sont transmis de génération en génération.

## Introduction

As young people continue to experience sexual and reproductive health (SRH) risks such as unplanned pregnancy and sexually transmitted infections (STIs), including HIV, much effort to improve their SRH, both in high- and low-income countries, has aimed to change the personal behaviours that put individuals at risk. Yet it has been widely recognised that risky behaviours are often driven by structural factors, that is, by the underlying patterns of social systems that are largely beyond an individual's control (Auerbach, Parkhurst, and Caceres 2011). In particular, individual choices regarding SRH are constrained by economic, legal, political, religious or other cultural factors at a macro level. If these structural elements remain unchanged, there is limited scope for changes of knowledge, norms, intentions or skills at an individual level to have much effect (Gupta et al. 2008; Padian et al. 2011).

There is considerable evidence that characteristics of families, and particularly parent-child relationships, have a major influence on young people's lives and sexual decision-making (Mmari and Blum 2009; Roche, Ahmed, and Blum 2008). We construe parenting as being not just individual behaviours, but a set of shared norms, beliefs, and practices that are institutionalised and therefore operate at a structural level. Previous publications by the first author have explored how specific dimensions of parenting and parent-child communication (Wamoyi et al. 2010, 2011c) and parental control and monitoring (Wamoyi et al. 2011c) affect young people's SRH.

This paper builds on these findings and uses wider data to show how overall patterns of parenting and family structure affect young people's SRH in a structural way. It starts by providing a conceptual framework and then outlines the qualitative methods. We then illustrate the structural influence of family context and parenting on young people's SRH, considering, first, how parents shape their children's abilities and behaviours and, second, how parents reproduce macro-structural factors affecting wider society, using gender as a specific example.

## Conceptual framework

**Figure 1:**
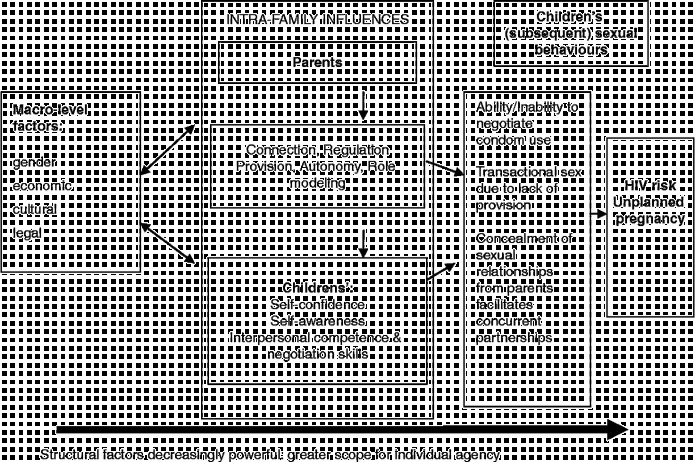
Structural influences of, and on, the family, and pathways to sexual behaviour.

Our focus here is on how the family acts in a structural way to impact on young people's vulnerability or resilience to poor SRH, although we acknowledge that macro-level structural factors also shape how families themselves operate (Figure 1). We conceive of structural factors as operating at different socio-ecological levels (Evans, Jana, and Lambert 2010) but, as indicated in Figure 1, it is important to acknowledge that as one moves from the micro to the macro level, structural factors become more powerful and allow less scope for agency.

Two different processes can be distinguished. First, within families, parents shape children's abilities and behaviours and, second, as the primary site of socialisation, the family reproduces the macro-structural factors affecting wider society. By ‘parent’ we mean to refer to any primary caregiver, including biological parents, grandparents, uncles and aunts and older siblings.

There are three main processes by which families and parenting may influence children's later sexual behaviour, evidenced primarily from research in high-income countries but increasingly confirmed in low-income countries. First, a nurturing environment high in warmth and low in hostility is likely to boost a child's confidence, self-awareness and emotional stability (WHO 2007). This, in turn, is likely to help a child think through their intentions for the future and identify their own sexual needs. Second, these personal attributes are essential to develop interpersonal skills and social competence during adolescence (Conger et al. 2000), for instance the ability to communicate needs and to assess other people's actions. This interactional competence has been shown to be associated with safer sexual behaviour (Vanwesenbeeck et al. 1999). Third, in early adolescence, parents' expectations for their children's behaviour, expressed through their own sexual values, their advice and rules for their children and their role-modelling (intentional or unintentional), all serve to influence their children's sexual behaviour (Oyefara 2005).

Both family structure, by which we refer to the composition of those with ongoing family relationships with each other, and parent-child interactions are highly affected by poverty. Parents are less likely to remain together if fathers have to seek work elsewhere or if material hardship exacerbates marital tensions (Babalola, Tambashe, and Vondrasek 2005; Borawski et al. 2003). Moreover, economic hardship often means that parents have less time to spend with their children due to long working hours, reducing their connection with their children and ability to regulate their behaviour (Babalola, Tambashe, and Vondrasek 2005; Borawski et al. 2003).

A second way in which families and, in particular, parents influence children at a structural level is as the main mechanism of socialisation (Rodger 1996). As parents raise their children they transmit socio-cultural beliefs and practices to a new generation. However, this process is filtered by their current structural environment (e.g., economic conditions and poverty), personal characteristics and social trajectories.

There is an intimate relationship between macro-level structural influences and family processes, since parents' parenting practices are shaped by wider structural factors and, in turn, reproduce them. Thus, parenting can be seen as a classic ‘social practice’ that produces, and is produced by, social structures (Giddens 1979). The main structural factors reproduced through families and shaping SRH-related vulnerabilities include gender relationships, generational hierarchies, economic poverty and cultural beliefs. All these structural factors manifest themselves in gender relationships, which are central to sexual health.

Gender norms influence parents' spousal relationships and shape how parents treat their children, for instance in the attention paid to them as infants, the allocation of domestic work or in restrictions on public activities. This leads children to acquire elements of gender identity from a very early age and shapes expectations of appropriate gender roles. In most societies, girls are socialised into accepting different forms of male superiority. This restricts their self-confidence, which, in turn, limits their ability to assert their interests in interactions with boys (Plummer and Wight 2011). When it comes to starting a sexual relationship, young people develop their sexual roles in terms of the gender identities they have previously learned, indeed, some argue that one of the main motivations of early sex is to develop and confirm one's own gender identity (Gagnon and Simon 1974).

An extreme example of how parents reproduce gender relationships is through the intergenerational transmission of domestic violence, with boys who witness domestic violence being far more likely to abuse their partners themselves (e.g., Martin et al. 2002). Since violence between sexual partners exacerbates the risk of HIV (Dunkle et al. 2004; Maman et al. 2000), this is one of the more direct ways in which harsh parenting can cause SRH problems. There are, of course, far more positive examples of parents' influence on gender relations (Warrington 2012)

The importance of gender and poverty as structural factors for poor SRH have already been well established (Parkhurst 2012; Pronyk et al. 2008). In this paper, we do not want to downplay their importance, but rather to show that family structure and parental upbringing can be seen as other important social determinants of SRH, whether positive or negative.

## Methods

### Design

The data reported on here come from an ethnographic study conducted in rural northern Tanzania in 2007 that explored the influence of families, parenting practices and socio-economic circumstances on young people's sexual behaviour. Data were collected using participant observation (PO), in-depth interviews (IDIs) and group discussions (GDs).

Combining these methods enabled greater understanding of complex issues related to parent-child relationships and young people's sexual behaviour. Participant observation provided data on everyday interactions within families and improved understanding of economic circumstances by experiencing them directly. Group discussions revealed community-level discourses about parent-child interactions, while IDIs solicited individual accounts of parent-child interactions.

Ethical approval was provided by the Tanzanian Medical Research Co-ordination Committee and permission to conduct the study granted at district, ward, village and participant levels. Oral and written consent was obtained from all participants. For those aged below 18 years (the age of majority in Tanzania), oral consent was also sought from parents or caregivers.

### Study setting

The study was conducted in the Kisesa demographic surveillance site in Mwanza Region, north-western Tanzania, where ‘remote’ rural villages account for 55% of the total population, roadside villages 16% and the central trading centre 28% (Magu Demographic Surveilance Site 2013). In Tanzania, primary school enrolment is between the ages of seven and nine (Bommier and Lambert 2000). Most young people complete primary school between the ages of 15 and 17, many having to repeat a year. Those who pass their primary school exams are expected to join secondary school, but there are several reasons why many do not, especially financial constraints. Recent national school enrolment rates in Tanzania were 94% for primary school and 35% for secondary school (UNICEF 2009). The few who complete the six years of secondary schooling typically leave in their early-20s (Plummer and Wight 2011).

### Characteristics of study participants

The participants were young people aged 14–24 years and parents and caregivers of that age group (Table 1). Twenty families from one village were selected for PO, with the help of the village authorities. Initially they were recruited from amongst those the researchers met and then through snowballing.

**Table 1:** Characteristics of study participants by IDIs and GDs.

	Male (*n* = 121)		Female (*n* = 105)	
Characteristics	IDIs (*n* = 21)	GDs (*n* = 90)	IDIs (*n* = 25)	GDs (*n* = 80)
Parents	9	30 in 3 groups	11	30 in 3 groups
Young people (14–24 years), total	12	60 in 6 groups	14	50 in 5 groups
Out-of-school	7	30 in 3 groups	9	30 in 3groups
In school (primary/secondary)	5	30 in 2 groups	5	20 in 2groups

Note: IDIs = in-depth interviews; GDs = group discussions.

Subsequently, purposive sampling ensured that the 20 families were representative of different family types in the community: 15 dual-parent (12 non-polygamous, 3 polygamous) and 5 single-parent families (1 single father, 4 single mothers). Of 46 IDIs, 25 were conducted with women (14 young women, 11 mothers/female caregivers) and 21 with men (12 young men, 9 fathers/male caregivers). Eight GDs were conducted with women (5 with young women, 3 female parents/caregivers) and 9 with men (6 young men, 3 male parent/caregivers). More than half of the young people interviewed had completed seven years of primary school and a small number were attending secondary school.

Both mothers and fathers were included in the study since we were interested in the interactions of both with young people and the potential role of both in SRH interventions. The population was predominantly Sukuma and Christian, while the principal economic activity was farming.

### Data generation

Data were collected by a Kenyan female graduate researcher (first author) and a Tanzanian Sukuma native male research assistant, a sixth form secondary school graduate. The first author's experience conducting extensive participant observation research in Mwanza, and both researchers' familiarity with local culture and their ability to speak at least limited Sukuma and fluent Swahili, facilitated interaction with participants.

Data collection commenced with PO. The two researchers resided with different families and visited the other 18 families several times over a period of eight weeks. Observations were conducted with the aid of a checklist to ensure that they were focused. Jottings were taken daily. At the end of the PO, GDs and IDIs were conducted with participants from seven villages including the PO village. Group discussions were conducted in two phases. For each group, three days were spent recruiting and getting to know pre-existing friendship groups to facilitate open discussion about sensitive issues. The GDs focused on broad issues related to parenting and young people's sexual behaviour. Group discussions with parents were organised by gender, while those with young people were also organised by gender and whether the young person was in or out of school. The second phase of GDs involved conducting two more GDs (1 with young women and 1 with young men) to explore issues that had emerged from preliminary analysis. Issues explored in the additional GDs were: gender relations in families, the role of gender on parenting practices, parental control and monitoring and power dynamics in sexual relationships.

Interviewees were selected from participants in the first phase of GDs to ensure variation by sex, schooling status and responses given during group discussions. This built on the rapport established during the GDs and explored at a personal level some of the issues that had emerged in the GDs. Initially, 36 interviews were conducted. After analysing the data generated, seven further interviews were conducted. Three of the parents were interviewed a second time to explore issues further, and another four interviews were conducted with new participants to explore issues that had emerged in the first interview phase.

 Group discussions and IDIs were conducted in Swahili, the national language of Tanzania. Data were collected on family structure, socio-economic status, material and social support and young people's sexual behaviour. For this paper, we analysed data on parenting practices, family interactions, the role of gender in parenting and young people's sexual behaviour.

### Analysis

Data were transcribed and translated into English by trained and experienced translators. Prior to importing documents into QSR NVIVO 7 software for coding, all the translated documents were quality checked to ensure no loss of meaning, with feedback sessions between researchers and translators. A pragmatic approach to coding combined prior codes, developed from the research objectives and prior knowledge, with codes that emerged through a thorough reading of the data and reflected participants' language and expression of ideas. Since research took place as part of a doctoral study, coding was done by the first author in close consultation with two co-investigators on the study. Thereafter, codes were developed into more conceptual categories and, finally, into themes. In presenting the findings, pseudonyms have been used and in the quotes ‘PO’ refers to participant observation, ‘I’ refers to the interviewer, while ‘R’ is the respondent.

## Findings

### Family structure and context of parenting

The majority of the young people reported that they lived with both parents, but the presence of parents in the household varied considerably. In some cases, one of the parents, usually the father, only appeared once in a while, and in others neither parent was physically present most of the day, for instance if the father was polygamous and the mother engaged in petty trade all day.

Six of the young people interviewed reported that they lived with grandparents: with both grandparents (*n* = 3), one grandparent and one parent (*n* = 2) or just one grandparent (*n* = 1). For some this was through force of circumstance: having been born out of wedlock, one or both parents having deceased, having had a disagreement with ones parents or ones parents living far away. For some, such living arrangements were through choice, with young people having decided to assist the grandparents with work or perceiving them to be less strict than their own parents.

According to the village chairman, 147 out of 709 households were single-mother headed, through widowhood, separation or never having been married. Household composition was not static and children could experience changes between single-parent and two-parent households.

Conversations with several participants showed that single-mother headed families were disadvantaged in many ways. Mothers were sometimes absent most of the day through selling their labour outside the home. They also lacked respect and generally had high levels of poverty compared to most two-parent families. Single-father households were rare and more temporary than single-mother households. Three single-father families were observed during PO, one resulting from the spouse's death, the other two from separation.

### Socio-economic context of young people's families

The main economic activities were subsistence farming and petty trade, while some laboured on others' farms or worked at the local stone quarry. There were more economic opportunities for young men than young women: for example, *daladala* businesses (bicycle taxi), brick making and house construction. The village microfinance scheme was open to married people with permanent residence in the village, which excluded single young mothers. Members paid a monthly contribution of Tsh 100 (0.06 US$) and could borrow up to the value of their household assets.

Most families seemed to struggle to get their basic needs. Typically, two meals were eaten each day, the first, of porridge or sweet potatoes and water, at around 3.00pm after farm work or school. The evening meal mainly consisted of *ugali* (stiff maize porridge) with green vegetables and beans or fish stew. Meat was usually restricted to special occasions (e.g. Easter), but meals varied with the seasons, with better diets during harvest season.

Most houses were of earthen walls and thatched roofs with one or two rooms. Small children under the age of five generally shared a sleeping house with their parents, while most young people slept in a separate house with same-sex siblings, on crude mattresses on the earth floor. Most young people had few clothes and wore plastic sandals, and old cloth was used for sanitary protection. Few households had a bicycle and about one-in-five a radio, generally owned by men. Household items were usually bought when required in very small quantities for single use. The village kiosks got busier in the evening, especially with demand for cooking oil and paraffin. The following was observed in one dual-parent household:

Jane's family purchased household supplies like cooking oil in small quantities as need arose. She shopped in small quantities because she could not afford to buy in bulk for several days use. (PO notes)

### Shaping children's abilities and behaviours

During infancy, and especially while breastfeeding, biological mothers did most of the childcare, including teaching children appropriate behaviour. Once the child had stopped breastfeeding, older siblings and other relatives took on more childcare since mothers were then more likely to be away from home for other activities. As children became more independent and started schooling, other non-resident relatives such as grandparents and aunts increasingly contributed to childcare.

The views of GD participants differed regarding children's behaviour and family structure. While most mothers thought that children in single-mother families had worse behaviour than those from dual-parent families, a few reported that family structure did not matter. Some fathers also thought that children's behaviour depended on parental upbringing, so strict single mothers could have better behaved children than lax dual parents. Ironically, mothers were more likely than fathers to think that women were not capable of ensuring good behaviour in their children.

It was rare to see parents spending their leisure time – something largely restricted to men – with their children. However, parents worked alongside their children, in domestic work and farming. Parental love was largely demonstrated through provision for material needs, but it was also expressed through mothers preparing meals with their children and then mothers eating with their daughters and fathers with their sons, during which time they might ask about their children's lives.

Cultural factors stipulating appropriate parent-child interactions and shaping how parents bonded with their children were interwoven with material factors. During the study, the researchers observed that some economic activities involved parents being away from home most of the day, leaving very little time to be with their children. Out-of-school young men reported:

Even the female children cannot stay at home because both parents are never around and you [male child] usually go for work … there is no one to control them. (GD)

### Respect for parents and interpersonal skills

From infancy, children were brought up to demonstrate respect to their parents and parents' generation, called *heshima.* This was characterised by unquestioning obedience and appropriate behaviour, especially in the presence of adults, in particular the *shikamoo* greeting, and being discreet about any extra-marital sexual activity. Children were expected to be largely quiet in the presence of adults and to only express their opinions after adults had first done so. Parents sometimes talked about creating an element of fear in order to achieve respectful children:

I decided to talk to them [children] so that they could fear me … I mean they can respect me. (IDI, 46-year-old single mother)

Highly confident children were seen as problematic and sometimes thought to lack *heshima*. Since proper *heshima* meant avoiding being seen as too self-assured, this probably affected young people's confidence and self-esteem, in turn affecting their competence to negotiate sexual relationships, for example, using condoms.

### Shaping sexual behaviour in early adolescence

The most direct way in which parents shaped their children's sexual behaviour was through their own sexual values and expectations for their children, expressed through advice and rules, material support and intentional or unintentional role-modelling. Parents were strongly aware of the consequences of their own behaviours on their children.

The way young people obtained their material needs tended to differ between the sexes and by schooling status. School boys and girls reported that most of their needs were met by their parents, while the majority of those out of school said they catered for most of their needs themselves, including basics like clothing. The rationale given by both parents and young people was that in-school children required more support to enable them to focus on their education. In many families, young people contributed financially to the daily needs of their families, particularly after leaving school. As one father commented:

We parents maintain them [children] but they maintain themselves too. Starting from the age of seven years … we are having problems getting their school needs or maintenance generally. (GD, fathers)

This changed the power relations in the family, greatly reducing parents' authority to control children's behaviour.

The less parents were able, or chose, to provide for their daughters, the greater incentive daughters had for sexual relationships, since they nearly always involved material exchange. Hence, most fathers mentioned that some parents prioritised their daughters' personal needs over their sons'. This was because they believed that their sons would tolerate hardship more than their daughters:

When a parent is in a good financial position, he thinks that if he wouldn't fulfill all the needs of his daughter, she could be tempted to join harmful groups and become pregnant or fail to study … but a male child can't be tempted. (GD, out-of-school young men)

Although parents tried to provide for their daughters' needs, their perceptions of what these were generally much more limited than those of their daughters. Parents generally only provided what they regarded as essentials, such as food, clothing and healthcare, and young people had to obtain other requirements, such as mobile phones or beauty products, themselves, for instance through petty trading, farming or transactional sex for young women:

… the important needs are shelter and medicine. Others are just additional. (GD, mothers)

The extent of parental regulation varied considerably with family structure, fathers' presence and parental occupation. Several occupations led one or both parents to be away from their families for long periods during the day, such as working at the rice mill, petty trade between the village and the nearby urban centres, stone quarrying and fishing.

Typically, daughters were only allowed out alone if doing domestic chores and had to be home by nightfall, while sons were able to play with their friends and were more likely to be allowed into the village centre in the evening, when it was an overwhelmingly male setting. Young people who generated their own income spent more time in the village centres in the evenings and had more scope for sexual relationships, both because of less surveillance and, for the young men, because they could pay for it. Some single mothers sent their daughters out to marketplaces in the evening to sell goods, requiring them to return home in darkness:

Maria is a 12-year-old-girl. She lives with her single mother and a 9-year-old. Every evening, her mother prepares *chapati* (shallow fried bread) for her to sell at the village market in the evening as her mother stays home to prepare dinner for the family. (PO notes)

### Parenting as a core mechanism in the reproduction of (other) structural factors

Parents sometimes transmitted to their children aspects of culture that promoted poor SRH. Examples of these were: the central place of sexual experience in masculine identity; concepts of feminine sexual respectability; the importance of maintaining discretion in sexual relationships, rather than strict adherence to moral norms; and negative beliefs about condoms. Other elements of culture reproduced by parents that affected young people's vulnerability in more general ways were a sense of limited agency and the related practice of short-term decision making.

### Gender norms

Most productive and social activities were segregated by gender, with the exception of very young children's activities. Men were generally more powerful than women, both socially and economically, having greater access to paid employment and the possibility of owning land and cattle. Men were generally assumed to be more knowledgeable than women, and any man in a household generally had greater authority than the women and was responsible for major decisions. Both parents and young people talked about fathers as having a strong economic interest in their daughters' sexual relationships since they would eventually receive bridewealth or fines for elopement or pregnancy. After marriage, young women normally moved to their husband's family home, sometimes in an entirely unfamiliar village, where they were expected to follow their in-laws' wishes.

Young people recognised that fathers were the overall decision makers in the family. For example, they mentioned that when they made a mistake it was usually their fathers who warned them, and when they wanted permission to do something they had to ask their fathers, either directly or through their mothers. Mothers were perceived as largely powerless:

I fear my father because he is the one who makes all the decisions. He can decide to forbid or allow you to do a certain thing, but my mother can forbid but if father allows me … then I just do it. (IDI, 20-year-old out-of-school boy)

In single-parent families, decisions about a daughters' marriage or the sale of family property were discussed and decided on by male relatives. This was because single mothers perceived such issues to be too big for them to handle and, moreover, these were traditionally not women's roles:

… he [suitor] came home and said, ‘mama I have come to be born here’ [presenting a marriage proposal] … I asked him, ‘…for which daughter?’ … ‘for your younger daughter Clare’. Then I told him, ‘My son, I cannot talk about these issues [marriage proposal] … I have a brother, maybe I should tell him to come. (IDI, 46-year-old single mother)

Participants talked about the general perception that single mothers could not enforce rules properly with their children. Parents said that female control of the family and children's behaviour was particularly frowned upon if men were present, in which case men were denigrated by the community. Ironically, most participants who thought that fathers should control families came from single-mother families themselves. Moreover, similar to participants from dual parent families, single mothers also valued sons more than daughters. Parents talked about single mothers lacking social respect because of their marital status and the possibility that respect could be enhanced if they had male children who could protect them and give them confidence in the future:

A male is respected more … because he is male…. For example, the way I live with my 17-year-old son, they [villagers] fear me because I have a male child …. They cannot do anything to me. (IDI, 40-year-old single woman).

These gender norms were reproduced through socialisation from a very early age. Men ate separately from women at home and fathers and sons were served food first. In the course of eating, the younger girls kept checking that the men had enough food. When the family chatted after a meal, young women rarely contributed and did not look at their male relatives in the face, which would be perceived as lack of respect:

While visiting one of the families, I observed that as we had a chat on general things, a young woman present (a niece to the family head) was uneasy. She did not contribute to the discussion. When food was served, she ate from inside the house. Their uncle later commented that his nieces could not seat next to him or talk in his presence, as that would be disrespectful. (PO notes, 30-year-old man)

Sons were monitored much less than daughters, and once they reached adolescence, contributed to family decision-making and were greeted with a deferential squat.

Parents were generally more willing to pay for their sons' education than their daughters', for several reasons, based largely on a realistic assessment of their children's prospects. Girls' were considered destined to marriage and domestic and farming work, for which education is unnecessary, while boys had more opportunities of paid employment. Some believed that their daughters' education would only benefit the family into which she married, whereas a sons' education would benefit them:

For the parent who has got ability to educate that child, she/he expects that when a male child gets a job he will support them. (IDI, 42-year-old man)

Furthermore, girls were thought likely to have unplanned pregnancies and drop out of school, wasting the resources devoted to education. Although most mothers and young women themselves thought it proper to prioritise boys' education over girls', some thought it important to invest in girls' education.

### Gender socialisation and SRH

These gender norms exacerbated women's poor SRH in several ways. First, girls were socialised into accepting male superiority, deferring to male decisions and seeing their role as serving men, while boys tended to be more self-assured, independent and assertive. This disadvantaged young women in sexual negotiation, both to resist unwanted sexual encounters and to insist on condom use. Young women's limited economic opportunities meant transactional sex played an important role in their lives, and once in established relationships their economic dependence eroded their scope to resist unsafe sex.

On the other hand, for young men, the central role of sexual experience in establishing their masculinity made them vulnerable to HIV and other STIs. Out-of-school young men said that when fathers were in a good mood, they sometimes talked about their sexual prowess with their sons.

Parents' communication about sexuality with their children was a further way in which gender roles were reproduced:

There is a day you are seated at home as a family, all happy …. Father jokes about how he used to attract girls when he was young … it is possible that the old man [father] has not seen you with a girl. He wants to assess your ‘sharpness’. (GD, out-of-school young men)

In so much as marriage reduces young women's SRH vulnerability, authoritarian parenting that emphasised the importance of marriage for daughters can be seen as protective for SRH. Many mothers brought up their daughters largely as they had been brought up, with the same focus on correct behaviour and the same ambitions and expectations that marriage would be the most important achievement in their lives. When mothers sat with their daughters, they talked about how they had abstained from sex until marriage and they expected their daughters to do the same. A key marker of good parenting of daughters was their marital eligibility, which was largely based on their reputation and respect for adults (*heshima*). Some women attributed their ability to get married to the strict parental upbringing they received:

My worry was that … my parents brought me up and educated me … now why can't I control myself and at least earn them two cows …. You see, I had to be careful while out there …. My father was also strict. (IDI, 35-year-old married woman)

Marriage brought income to the family through bridewealth, leading parents to emphasise the need for their daughters' ‘good behaviour’:

A girl is business … she is a commodity for her family. (GD, male parents).

## Discussion

These findings highlight the structural influence of family composition and parenting on young people's SRH. Although the data collected do not allow us to show precise relationships between family factors and young people's sexual behaviour, we can draw on participants' accounts of influences and infer from the literature how the observed parenting norms and practices are likely to affect young people's vulnerability or resilience. This is through shaping their abilities and behaviours and by reproducing other structural factors that create vulnerability, in particular gender relations and cultural values about sexuality.

Family structure is also important in determining young people's sexual behaviour. There is increasing evidence from sub-Saharan Africa of the association between parents' presence in the household and young people's sexual behaviour (Ngom, Magadi, and Owuor 2003). It is likely that parents' presence has a protective influence on their children in several different ways, for instance, initially by strengthening parent-child connections and later by allowing greater parental monitoring (Wamoyi et al. 2011a). For example, in Uganda, young people reported that they were sexually active when their parents were away or when they were staying with other relatives (Twa-Twa 1997). The importance of parental presence should encourage policy makers to pay more attention to the socioeconomic factors that lead parents to spend long periods away from home.

We found that many co-resident parents had limited time with their children and, in particular, many fathers were rarely present in the household. This suggests that, from an early age, children's confidence, self-awareness and emotional stability were likely to be shaped as much by their siblings, other relatives and peers as by their parents. Fathers' absence and children's deferential relationship with their parents meant that children's interpersonal skills were probably learnt more from their siblings and peers than from their parents. We have limited data on the effect of this, but it seems unlikely to have helped children develop mature ways of negotiating and resolving conflict. This limited interpersonal competence would, in turn, impact on their sexual negotiation and decision making (Vanwesenbeeck et al. 1999).

Rapid and profound social changes are affecting families in Tanzania, as elsewhere in sub-Saharan Africa. These include higher levels of education for children (especially girls); greater knowledge about SRH matters; young people increasingly contributing materially to the household; and parents working for long periods away from their children (Wamoyi et al. 2011b). These changes make the parent-child relationships that socialised children into agricultural lifestyles increasingly inappropriate and make parents more conscious of their parenting roles. The changes have been both positive and negative.

Parents' ability and readiness to provide materially for their daughters have important implications for their SRH, as other studies have shown (e.g., Plummer and Wight 2011). Notable among these are young women engaging in transactional sex (Longfield 2004; Wamoyi et al. 2010), which has been associated with unsafe sex and other undesirable sexual health outcomes (Jewkes et al. 2012). Parents are increasingly unable to meet the consumer demands of their children, which the latter have come to regard as essential to social life (Wamoyi et al. 2011b).

Interventions to improve young people's SRH should recognise the structural effects of parenting, both in terms of direct influences on children and through reproducing structural barriers such as gendered power relations and cultural norms around sexuality. An ecological approach to such interventions would attempt to impact on the changing socioeconomic landscape of parenting at different levels. For example, facilitating new means of income generation could help parents provide for their children materially.

More directly, the impact of norms around *heshima* on young people's self-confidence and interpersonal competence need to be addressed. Parenting programmes could assist parents to identify positive parenting practices that foster healthy adolescent development, for example by expanding concepts such as *heshima* to incorporate the value of their children having self-confidence in life. Such programmatic interventions are likely to have generic development benefits, given that more authoritative styles of parenting are likely to foster the kinds of social competences necessary to succeed in more economically developed societies (Baumrind 1978).

## Conclusion

In considering how best to promote young people's sexual health in contexts such as those described here, we should address more fully the family context and young people's relationships with their parents. Interventions that fail to acknowledge the structural influence of parenting and family context and focus solely on developing young people's knowledge, attitudes and skills are likely to only have a minimal impact in reducing young people's vulnerability to poor SRH.
